# Chemically modified aptamers for improving binding affinity to the target proteins *via* enhanced non-covalent bonding

**DOI:** 10.3389/fcell.2023.1091809

**Published:** 2023-02-23

**Authors:** Zefeng Chen, Hang Luo, Amu Gubu, Sifan Yu, Huarui Zhang, Hong Dai, Yihao Zhang, Baoting Zhang, Yuan Ma, Aiping Lu, Ge Zhang

**Affiliations:** ^1^ Law Sau Fai Institute for Advancing Translational Medicine in Bone and Joint Diseases, School of Chinese Medicine, Hong Kong Baptist University, Kowloon, Hong Kong SAR, China; ^2^ Institute of Integrated Bioinfomedicine and Translational Science, School of Chinese Medicine, Hong Kong Baptist University, Kowloon, Hong Kong SAR, China; ^3^ Aptacure Therapeutics Limited, Kowloon, Hong Kong SAR, China; ^4^ School of Chinese Medicine, Faculty of Medicine, The Chinese University of Hong Kong, Hong Kong, Hong Kong SAR, China; ^5^ Institute of Precision Medicine and Innovative Drug Discovery, HKBU Institute for Research and Continuing Education, Shenzhen, Hong Kong SAR, China

**Keywords:** aptamer, chemical modification, high affinity, non-covalent bonding, interaction

## Abstract

Nucleic acid aptamers are ssDNA or ssRNA fragments that specifically recognize targets. However, the pharmacodynamic properties of natural aptamers consisting of 4 naturally occurring nucleosides (A, G, C, T/U) are generally restricted for inferior binding affinity than the cognate antibodies. The development of high-affinity modification strategies has attracted extensive attention in aptamer applications. Chemically modified aptamers with stable three-dimensional shapes can tightly interact with the target proteins *via* enhanced non-covalent bonding, possibly resulting in hundreds of affinity enhancements. This review overviewed high-affinity modification strategies used in aptamers, including nucleobase modifications, fluorine modifications (2′-fluoro nucleic acid, 2′-fluoro arabino nucleic acid, 2′,2′-difluoro nucleic acid), structural alteration modifications (locked nucleic acid, unlocked nucleic acid), phosphate modifications (phosphorothioates, phosphorodithioates), and extended alphabets. The review emphasized how these high-affinity modifications function in effect as the interactions with target proteins, thereby refining the pharmacodynamic properties of aptamers.

## 1 Introduction

Aptamers are ssDNA or ssRNA fragments, which are typically screened from oligonucleotide pools using the systematic evolution of ligands by exponential enrichment (SELEX) technology. Because of the three-dimensional shapes, aptamers enable to recognition of abundant targets like antibodies (Chen and Yang, 2015). Aptamers take many advantages over antibodies, including easier synthesis, less time and cost consumption, lower immunogenicity, higher stability, and superior re-foldability. Hence, aptamers are promising substitutes for homologous antibodies. In 2004, the United States Food and Drug Administration (FDA) approved the first commercialized aptamer drug (Macugen^®^) that binds vascular endothelial growth factor protein 165 (VEGF165) for the wet-form neovascular age-related macular degeneration (AMD) (Ruckman et al., 1998; Rothlisberger and Hollenstein, 2018). Nowadays, several therapeutic aptamers have entered phase II-III clinical trials.

In ionic environments, aptamers enable the folding of numerous 3-dimensional (3D) motifs, e.g., hairpins, bulges, loops and pseudoknots (Strehlitz et al., 2012). It permits aptamers to form different shapes to interact with the ligands through non-covalent bonding, including hydrogen bonding, π–π stacking, London dispersion forces, ion-ion interactions, and dipole-dipole interactions. It is critical for aptamers to tightly interact with the target ligand (usually protein) to obtain high binding affinity (Hu et al., 2022). Nevertheless, aptamers should form a 3D structure for target recognition, possibly impeding the amplification efficiency during polymerase chain reaction (PCR). It provides a mechanistic insight involved in the undesired binding affinity of the resultant aptamers screened by SELEX to targets (Hasegawa et al., 2016). More importantly, natural DNA aptamers are composed of naturally occurring 4 deoxynucleotides (A, C, G, T), while natural antibodies are composed of 20 amino acids. Hence, the chemical variety of DNA aptamers is much more restricted in comparison with those of antibodies. Typically, naturally occurring aptamers show a decreased binding affinity than cognate antibodies. It indicates that the interactions between the aptamer and its ligand protein have spaces to be optimized.

**FIGURE 1:**
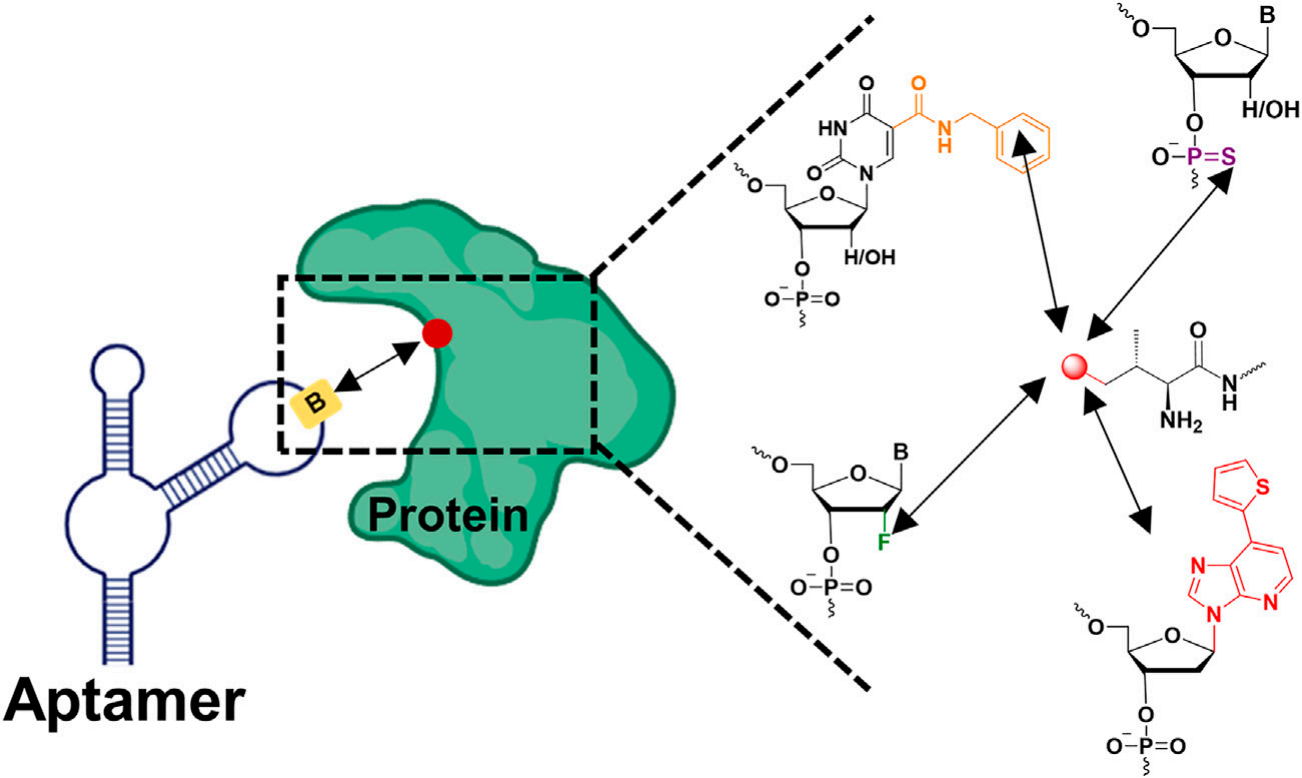
Chemically modified aptamers for improving binding affinity to the target protein *via* enhancing non-covalent bonding (the red dot represented amino acid residues on the protein, the yellow rectangle represented modified nucleotides on the aptamer, the black arrow represented non-covalent bond).

Researchers sought to determine whether chemical modifications could facilitate interactions between the modified aptamer and its ligand protein, with no increase in off-target effects (Lipi et al., 2016) (Figure 1.). Totally, the interactions can be classified as non-covalent and covalent, respectively. Although covalent bonding enhances the highest affinity, the miserable off-target effects demonstrate reduced therapeutic or diagnostic applications in aptamers. In contrasts, non-covalent bonding embodies the properties of high affinity and specificity. The absence of interactions of aptamers to targets can be remedied with modifications at either the nucleobase, sugar ring or phosphate backbone. The naturally occurring aptamers have reached a plateau in the treatment of diseases, and there is an urgent sense that affinity enhancement must now come from fresh modification approaches, such as hydrophobic groups, amino acids, positive charges and phosphorothioates. Importantly, expanding aptamer epitopes, *i.e.*, increasing the contact area between the modified aptamer and protein, largely contributes to the affinity enhancement (Zon, 2022).

In this review, we summarized an outlook of chemically modified aptamers for improving binding affinity to the target protein *via* enhanced non-covalent bonding. It will help guide the next-generation of chemically modified aptamers with high affinity for disease diagnosis and treatment.

## 2 Strategies for improving the binding affinity of the modified aptamer to the target protein *via* enhanced non-covalent bonding

### 2.1 Nucleobases modifications

#### 2.1.1 Nucleobases with amino acid-like side chains

Nucleobases can be modified with a wide variety of functional groups to improve the affinity between aptamer and protein. Given the inherent properties of nucleic acids and proteins nucleobases with amino acid-like side chains could confer further advantages to modified aptamers, because those amino acid side chains can directly participate in the interaction with protein.

**FIGURE 2:**
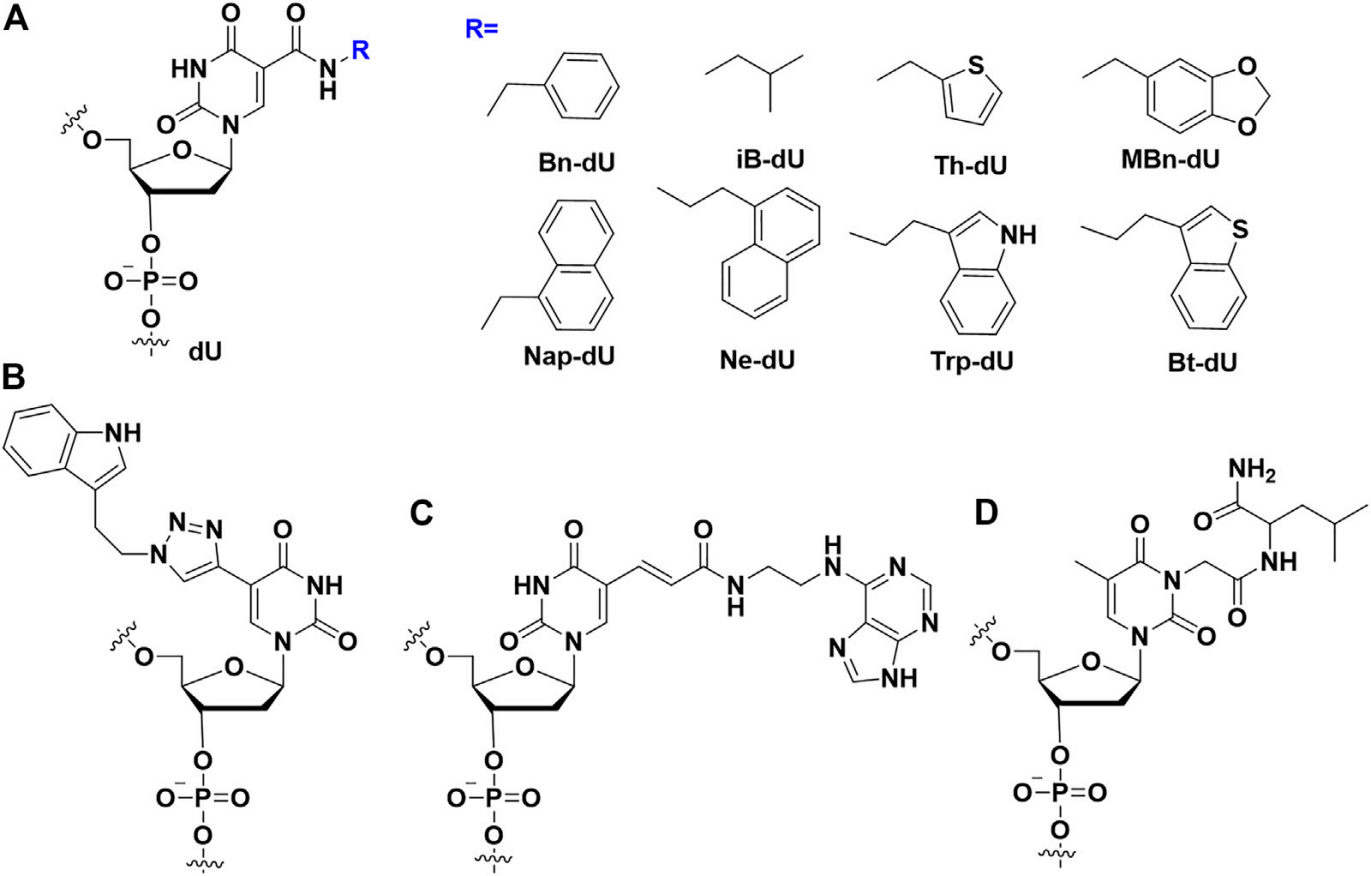
The nucleobases modifications on the aptamers to enhance binding affinity. **(A)** SOMAmers modification; **(B)** Modified uridine by click chemistry; **(C)** Base-appended bases modification; **(D)** Amino acid (leu) modification.

By modifying the C5 position of deoxyuridine triphosphate (dUTP) with some hydrophobic groups (Figure 2), Gold et al. combined the conformational flexibility of nucleic acids with the functional variety of proteins, which greatly enhanced the binding affinity of modified aptamers to proteins (Davies et al., 2012). dUTP modification at C5 can form additional hydrophobic interactions with hydrophobic pockets of proteins. These aptamers embodied slow off-rate to proteins, and were termed as SOMAmers (slow off-rate modified aptamers). Nebojsa et al. expanded nucleobases with amino acid-like side chains from uracil to cytosine. It was found that aptamers comprising modified uracil and cytosine exhibited higher binding affinity than that comprising modified uracil (Gawande et al., 2017).

Different from the SOMAmers in which an amido linker was used between hydrophobic group and dU, Günter Mayer et al. developed triazole linker by click chemistry between alkyne-modified uridine (5-ethynyl-deoxyuridine (EdU)) instead of thymidine and hydrophobic group-azide (Figure 2B). Combining the compatibility of EdU to DNA polymerase in SELEX procedure and highly efficiency of click chemistry, they succeeded to screen an indole modified aptamer against cycle 3 GFP with 18.4 nM of K_D_ value (Tolle et al., 2015; Pfeiffer et al., 2018).

Apart from hydrophobic modifications, hydrophilic modifications may facilitate forming additional hydrogen bonds and ion-ion interactions. Buyst et al. conjugated histamine to C5 of the thymine base in the aptamer by amido linkages (Buyst et al., 2015). The single modified thymine was placed at four different positions in the center of the 14mer double helix, from which the interaction between imidazole and double chain and its influence on imidazole pKa were studied. A structural motif is established by unrestricted molecular dynamics and nuclear magnetic resonance, involving the formation of hydrogen bonds between imidazole and the Hoogsteen side of two adjacent GC base pairs, which has been shown to significantly enhance the DNA Thermal stability of double strands.

It was reported that base modifications contribute to the thermal stability of synthetic DNA duplexes (Verdonck et al., 2018). Unconstrained molecular dynamics (MD) simulation showed that attributable hydrogen bonds could be formed between imidazole and the nearby guanosine. A principal validation study showed that the imidazole thymine modification could enhance the stability of L-arginine amide conjugated aptamers, indicating a vital role in stability enhancement. Park’s group demonstrated that the hydrophobic amino acids in aptamers could be regarded as a novel kind of unnatural nucleotide (Yum et al., 2021). They synthesized a series of modified thrombin aptamers (TBA) to investigate their affinity and antithrombin activity. Then found that the incorporation of amine acids could improve the binding affinity of TBA (K_D_ = 2.94 nM), and make the 3-fold of antithrombin activity enhancement.

#### 2.1.2 Base-appended base modifications

Horii and Waga’s group developed the base-appended bases (Figure 2C) to enhance the binding affinity of modified aptamers (Minagawa et al., 2017). Using modified SELEX, a high affinity aptamer could be isolated after several rounds of selection. For example, a Salivary α-amylase (sAA) aptamer (AMYm1) was selected, which had high affinity (K_D_<1 nM) with sAA. Ikuo et al. designed an adenine-appended modification strategy, and successfully screened an aptamer A^ad1^ against human β-defensin 2 (Minagawa et al., 2020) with the K_D_ value of 6.8 nM. Recently, Horii’s group applied base-appended modification strategy to select SARS-CoV-2 aptamer (Minagawa et al., 2022). These aptamers had extremely low K_D_ value (1.2 nM and <1 nM) to the receptor-binding domain (RBD) and spike trimer of the SARS-CoV-2, respectively.

#### 2.1.3 Other nucleobase modifications

Apart from above modifications, Smirnov et al. introduced amino acids, such as Leu (Figure 2D), to the nucleobase of TBA *via* post-SELEX modification (Smirnov et al., 2021). They demonstrated that these modifications could improve the binding affinity and enhance anticoagulant activity. The crystal data demonstrated that modified bases increased the contact areas between the target proteins and modified aptamers. (Cheung et al., 2020b). Cheung et al. report the affinity of cubane-modified aptamers (cubamers) for the malaria biomarker *Plasmodium vivax* lactate dehydrogenase (PvLDH) (Cheung et al., 2020a). Analysis of the crystal structure of the complex reveals a binding mechanism involving multi-carbon clusters within the hydrophobic pocket. SPR analysis of the binding affinity of cubamer to *Plasmodium falciparum* lactate dehydrogenase (PfLDH) and PvLDH, respectively, showed that cubamer had higher affinity and specificity to PvLDH. β-secretase 1 (BACE1) is the therapeutic target of Alzheimer’s disease, so developing a DNA aptamer that binds to BACE1 to treat the disease may be an effective measure. Herdewijn’s team added triphosphate analogs modified by 5-chlorouracil and 7-deazaadenine bases during the SELEX process to obtain high-affinity aptamers for BACE1 (Gasse et al., 2018). The finally screened aptamers showed significant affinity (K_D_ = 10 nM) to BACE1 with IC_50_ values in the low nanomolar range.

### 2.2 Ribose modifications

#### 2.2.1 2′-Fluoro nucleic acid (2′-F RNA)

**FIGURE 3:**
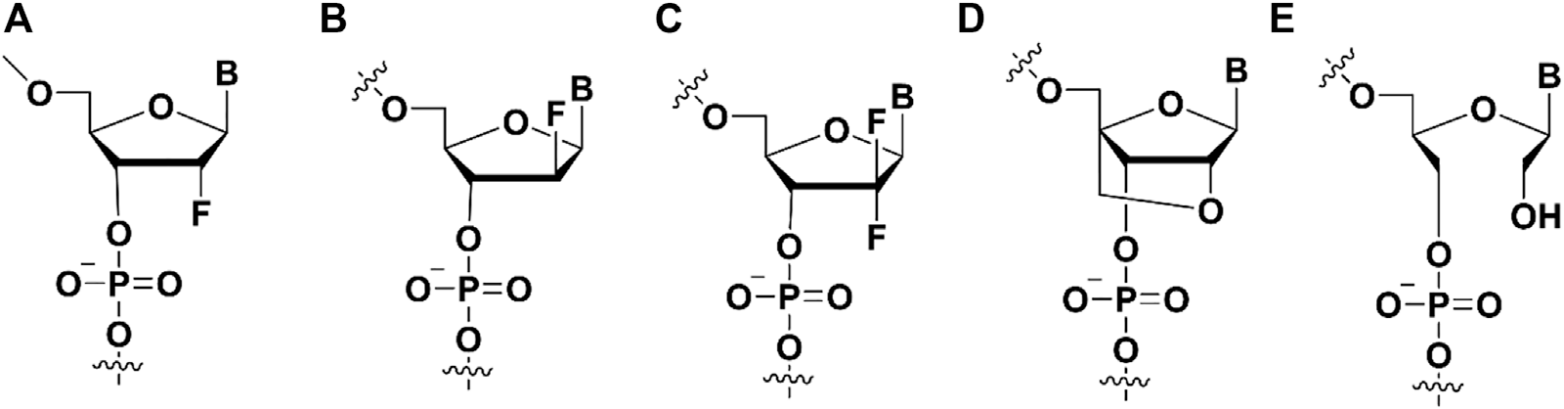
The ribose modifications on the aptamers to enhance binding affinity. **(A)** 2′-fluoro (2′-F) modification; **(B)** 2′-Fluoro Arabino nucleic acid modification; **(C)** 2′, 2′-difluoro-2′-deoxycytidine modification; **(D)** Locked Nucleic Acid modification; **(E)** Unlocked Nucleic Acid modification.

Modifications on the ribose part of nucleic acid, such as 2′-fluoro (2′-F), 2′-Fluoro Arabino, 2′, 2′-difluoro-2′-deoxycytidine (Figure 3), could enhance the binding affinity. Generally, unmodified aptamers have limited affinity to targets, and the 2′-fluoro (2′-F) optimized aptamers exhibit superior binding affinity and nuclease resistance (Maio et al., 2017; Odeh et al., 2020) (Figure 3A). For instance, oligonucleotides modified with 2′-F, but not with 2′,4′-constrained 2′-O-ethyl (cEt) or 2′-O-methoxyethyl (2′-MOE), exhibit higher affinity to 54 kDa nuclear RNA- and DNA-binding protein (P54nrb). Wen et al. isolated antisense oligonucleotides (ASO) binding protein from HeLa cell extracts using a biotinylated gapmer phosphorothioate (PS)-ASO with flanking nucleotides 2′-modified with F (ISIS623496), and competitively eluted it with non-biotinylated gapmer PS-ASOs of the same sequence modified on the flanking nucleotides with 2′-MOE, cEt or 2′-F (Shen et al., 2015). The affinity-selected proteins analyzed by western blotting showed that more P54nrb as well as fused in sarcoma (FUS) were preferentially eluted by 2′-F modified competitor ASO, rather than cEt or 2′-MOE-PS-ASO. Then, the author designed two kinds of gapmer oligonucleotides with 2′-F modification on 5′or 3′flanking nucleotides (called “wings”) and 2′-MOE modification on the other wings. These two PS-ASOs were used to elute ASO binding proteins coprecipitated by biotinylated 2′-F-PS-ASO. The separated proteins were then characterized through Western blotting. It was found that P54nrb as well as FUS had higher affinity for ASO with 2′-F modification on oligonucleotide 3′-wing.

#### 2.2.2 2′-Fluoro arabino nucleic acid (2′-F-ANA)

The sugar ring of 2′-deoxy-2′-fluoro-arabinonucleic acid (FANA) (Figure 3B) typically adopts a C2′/O4′-endo conformation rather than the C3′-endo conformation of 2′-fluorinated ribonucleotides (El-Khoury and Damha, 2021; McKenzie et al., 2021), the fluorine group of FANA is the β conformation rather than α (Trempe et al., 2001; Martin-Pintado et al., 2013). Recently, 2′F-ANA modified aptamers against HIV-1 integrase (IN) have been selected through SELEX (Rose et al., 2019). The dissociation constants of 2′F-ANA modified aptamers in the range of 50–100 p.m. were obtained, of which the K_D_ value exceeded two orders of magnitude than traditionally than unmodified DNA and RNA aptamers. The substitution of 2′F-ANA nucleotides significantly reduce the binding affinity. It indicates that the distinctive structural conformation of 2′F-ANA nucleotides in the aptamer is essential to improve the affinity.

Compared to unmodified thrombin aptamer (TBA), FANA modified TBA showed significantly higher affinity and nuclease resistance (Peng and Damha, 2007; Watts and Damha, 2008). Huang et al. drew a binding affinity map of 2′F-ANA modified aptamer with TBA (Lietard et al., 2017). A total of 32768 sequences were prepared on the microarray to plot possible mutations in DNA-to-2′F-ANA. Since 2′F-ANA was linked to tight interactions, the antiparallel folded G-quadruplex with 2′F-ANA modifications in loops had a profitable effect on binding affinity improvement. This work enhanced the potential aptamer research of 2′F-ANA and further expanded the non-genomic applications in the field of nucleic acid microarrays.

#### 2.2.3 2′,2′-Difluorocytidine (dFdC, gemcitabine)

Gemcitabine [2′,2′-difluoro-2′-deoxycytidine (dFdC)] (Figure 3C) has a fluorine substituent in the pentose ring, with involvement of the most important deoxycytidine analogs (Heinemann et al., 1988). As a pyrimidine antimetabolite and a prodrug, dFdC has been approved for the treatment of breast cancer, bladder cancer, ovarian cancer, colon cancer, non-small cell lung cancer and pancreas cancer (Dubey et al., 2016). Gemcitabine could be internalized into cells *via* nucleoside transporters (hENT2, hENT1) as well as concentrated nucleoside transporters (hCNT3, hCNT2 and hCNT) (Mini et al., 2006).

Previous studies had shown that the loop region in the G-quadruplex played a crucial role in nucleolar protein binding. Thus, chemical modification in the loop region might change the binding ability of the modified aptamer to protein (Kotula et al., 2012). Kang’s team has constructed a gemcitabine-modified AS1411 (APTA-12) (Park et al., 2018). The binding affinity data exhibit that gemcitabine modification (K_D_ = 14.37) could sightly enhance the binding ability of modified AS1411 to nucleolin (K_D_ = 16.36).

#### 2.2.4 Locked nucleic acid (LNA)

Locked Nucleic Acid (LNA) is a nucleic acid analog whose the conformation structure of the locked nucleic acid (LNA) is “locked” by a methylene bridge connecting the 2′-oxygen and the 4′-carbon atoms. The fixed C3’ internal conformation of LNA endows the modified aptamers with enhanced thermal stability, improved nuclease resistance, and stable base-pairings (Figure 3D) (Koshkin et al., 1998).

LNA is the most promising modified nucleotide widely used in antisense oligonucleotides, siRNAs and aptamers. Its characteristics in binding affinity and nuclease resistance are being extensively studied. Birte et al. described the changes in affinity after the incorporation of 2′-amino LNA and LNA monomers into avidin protein binding DNA aptamers (Hernandez et al., 2009). The kinetic curves of the selected modified aptamers were obtained by SPR and compared to those of the unmodified DNA aptamers. The results showed that LNA modified a new avidin aptamer, and the affinity was increased by 8.5 times. The incorporation of 2′-amino LNA served as a new monomer into the aptamer can also obviously improve the binding affinity of the antibiotin, and has the potential function as an additional molecular entity carrier unit.

#### 2.2.5 Unlocked nucleic acid (UNA)

As an acyclic analog of RNA, the bond linking to the C2’ and C3’ atoms in UNA (unlocking nucleic acid) (Figure 3E) ribose ring is cut off (Nielsen et al., 1995). In 1995, researchers first synthesized UNA thymine monomer, which was proven to significantly reduce the thermal stability of DNA double strands. Recently, the synthesis of UNA-A, -C, -U and -G phosphoramides, brought all UNA monomers into DNA and RNA double stranded bodies, and analyzed their thermodynamic stability (Campbell and Wengel, 2011).

The initial idea of UNA was to increase the flexibility of aptamer structures for promoting an induced fit mechanism between aptamer and target. As a 31 nt DNA aptamer, RE31 is composed of G-quadruplex as well as double stranded domains, which can effectively prolong thrombin time. Anna et al. reported the effect of some modified nucleotide residues on the thermodynamic as well as biological characteristics of RE31 and the changes in folding topology (Kotkowiak et al., 2019). In particular, the impact of nucleosides in unlocked nucleic acid (UNA) sequences was assessed. SPR spectroscopy was used to determine the interaction intensity between RE31 variant and thrombin. O3 containing a UNA-C residue at the T15 position obtained the most favorable K_D_ value (K_D_ value of 0.43 nM) compared with the unmodified RE31 (with a K_D_ value of 1.34 nM). Using UNA-A, UNA-U and UNA-G to modify the T15 position of the RE31 variant, the K_D_ values measured are higher but still lower than the unmodified RE32 (K_D_ values of 0.76, 0.68 and 0.75 nM for O2, O4 and O5, respectively). The results demonstrate that both UNA residues as appropriate molecular tools can regulate RE31 thermal as well as biological stability. Their results offer new views into the application field in modified DNA aptamers as potential replacements for classic antithrombin drugs.

#### 2.2.6 Xenogenic nucleic acids (XNAs)

**FIGURE 4:**
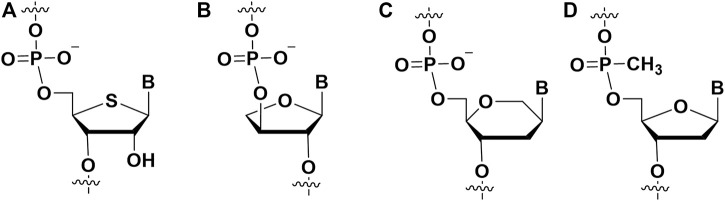
The ribose modifications on the aptamers to enhance binding affinity. **(A)** 4′-S modification; **(B)** TNA modification; **(C)** HNA modification; **(D)** phNA modification.

Nucleic acid backbone modifications can reduce its susceptibility to nucleases, and these modified nucleic acids can be used for targeting and therapeutic purposes. These nucleic acid backbone structures are significantly different from natural nucleic acids and are often referred to as xenogenic nucleic acids (XNA) (Meek et al., 2016). In the early 2000s, a research group introduced a modification that the 4′-oxygen atom of the sugar unit was replaced by a sulfur atom (Figure 4A) (Kato et al., 2005). 4′-Thioribonucleoside proved to facilitate enhancing the stability of modified aptamer without loss of the protein binding ability (Hoshika et al., 2004). For instance, Kato et al. used 4′-thioribose for the synthesis of 4′-thiouridine (4′-thioUTP) and 4′-thiacytidine (4′-TTP) triphosphate, which were then used for *in vitro* screening of anti-thrombin thioRNA aptamers (Kato et al., 2005). The resulting 4′-thio-modified aptamer bound the thrombin target with high affinity (K_D_ = 4.7 nM) and showed a 50-fold increase in resistance to RNase A compared to wild-type RNA. In addition, Noriaki et al. selected 4′-thioRNA aptamers by optimizing the nucleoside triphosphates (NTPs) concentration, and the most potent aptamers generated by this selection experiment showed high affinity binding to thrombin (K_D_ = 7.2 nM) (Minakawa et al., 2008).

Threose nucleic acid (TNA) (Figure 4B) as a type of XNA, the natural ribose in its RNA is replaced by unnatural threose. McCloskey et al. integrated chemically modified uracil residues into the 3′,2′-α-l-threofuranosyl nucleic acid library and screened out biologically stable TNA aptamers (McCloskey et al., 2021). The modified and unmodified S1 aptamers had K_D_ values of 3.1 ± 1.0 and 34 ± 11 nM for the S1 protein in SARS-CoV-2, respectively. Chaput’s team developed a TNA heterologous nucleic acid system that is significantly resistant to nuclease digestion (Dunn et al., 2020). Functional TNA aptamers can be isolated by engineered TNA polymerase with K_D_ values for HIV reverse transcriptase (HIV RT) ranging from 0.4 to 4.0 nM. This TNA aptamer has the ability of high binding affinity and thermal stability, which is a powerful system for developing diagnostic and therapeutic drugs. Among the other XNAs aptamers, three structurally unique 2′-O-methyl-ribose-1,5-anhydrohexitol nucleic acid (MeORNA-HNA) (Figure 4C)aptamers targeting rat VEGF164 target protein were successfully screened by the researchers (Eremeeva et al., 2019). The HNA/2′-OMe modified aptamer was able to bind rat VEGF164 with low nanomolar affinity (K_D_ = 1.1 nM). Additionally, Arangundy-Franklin et al. report the synthesis and reverse transcription of uncharged P-alkyl phosphonate nucleic acids (phNA) (Figure 4D) under full substructure using engineered polymerase variants (Arangundy-Franklin et al., 2019). The experimental results showed that the affinities of phNA aptamers T1-20 and T5-20 to streptavidin (SA) appeared in the low millimolar range.

#### 2.2.7 Spiegelmer modifications

**FIGURE 5:**
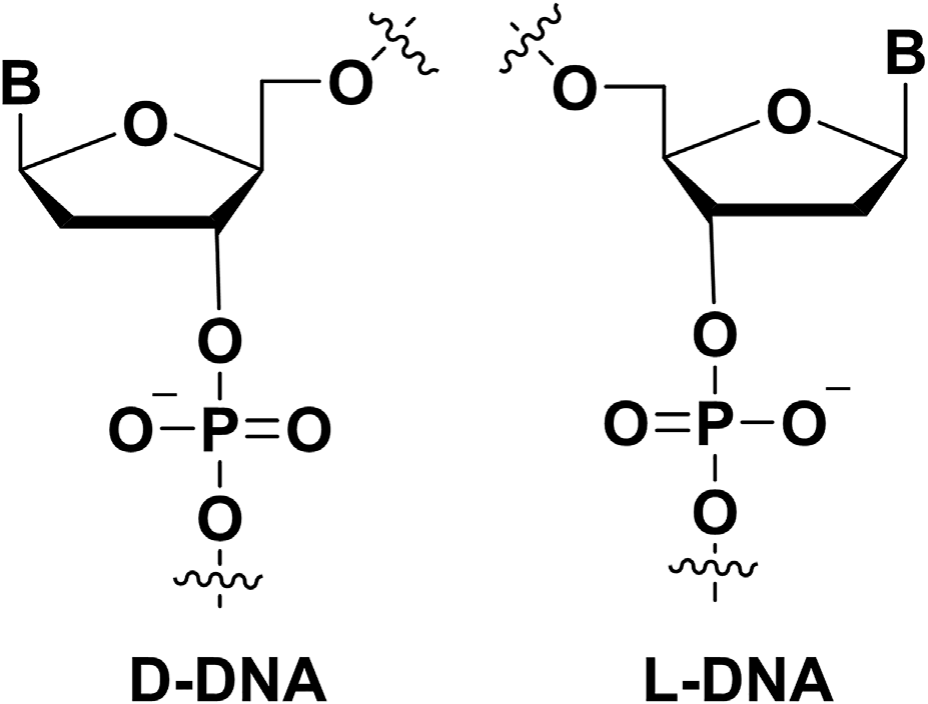
The mirror-image nucleotide (L-DNA) modification on aptamers.

Different from the chirality centers of ribose sugars in natural aptamers, the Spiegelmer (mirror-image aptamer) (Figure 5) have inverted chirality centers in ribose sugars. Because nature enzymes did not recognize L-nucleotides, it made Spiegelmer more stable in plasma. It is helpful to design the aptamer with good biostability. In the development of Spiegelmer, the critical point was that requiring the enzymes to recognize the L-nucleotides because the enzymes were necessary for the PCR or sequencing process. Zhu’s group developed the technology for the selection L-aptamers with mirror-image DNA polymerase (Chen et al., 2022). They selected the L-aptamers that could be used in inhibiting and detecting thrombin in nuclease-rich environments. Furthermore, there are many high binding affinity L-aptamers were generated in these years (Vater and Klussmann, 2015). For example, an important Spiegelmer, NOX-A12, could bind stromal-cell-derived factor-1 with a K_D_ of 0.2 nM. NOX-A12 was further applied in clinical trials with good therapy effect (Vater et al., 2013). In addition, other Spiegelmers also were evaluated in phase trials because its safety profile and therapy effect (Vater and Klussmann, 2015). Klussmann group developed the mirror-image aptamer (NOX-D19) to target complement factor C5a with a K_D_ of 1.4 nM (Hoehlig et al., 2013). The NOX-D19 also had therapy effect on animal model. In addition, a L-RNA aptamer was selected to bind D-RNA by Sczepanski′s group (Dey and Sczepanski, 2020), it expanded the application of Spiegelmer.

### 2.3 Phosphate modifications

#### 2.3.1 Phosphorothioate (PS)

**FIGURE 6:**
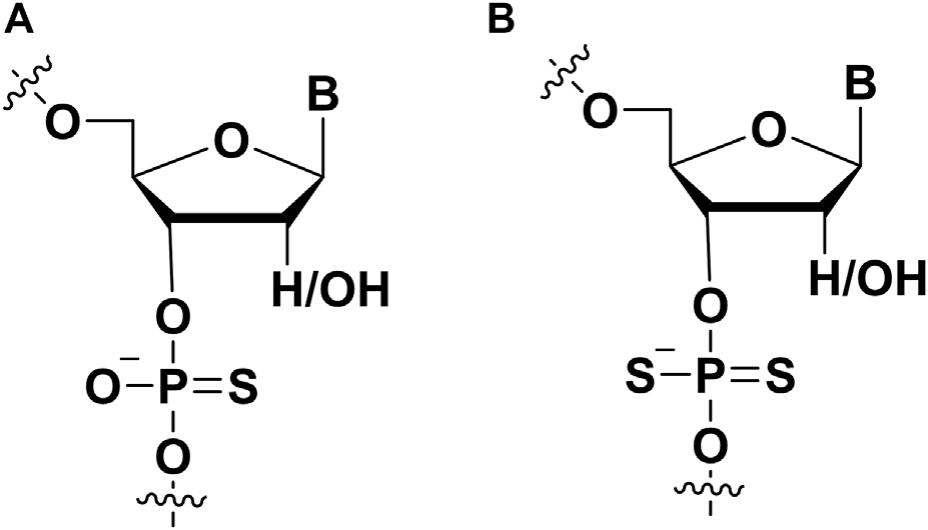
The chemical modifications on the phosphate of aptamers to improve affinity. **(A)** Phosphorothioate (PS) modification; **(B)** phosphorodithioate (PS2) modification.

Modifications on the phosphate part of aptamer are regarded as an important strategy to improve affinity, and this modification also can help aptamer to resist nuclease *in vivo*. Two non-bridging oxygens can be replaced by sulfur to form thiophosphorus (PS) and phosphorodithioate (PS2) (Figure 6). PS-modified DNA had been well established through solid phase synthesis (Blackburn et al., 2006). To synthesize PS DNA, the vulcanization step replaced the oxidation step (Saran et al., 2021). Vulcanization of newly formed phosphite triester ester with Beaucage reagent. This reaction would form two isomers (Blackburn et al., 2006; Frederiksen and Piccirilli, 2009). It was worth noting that single PS modification could be separated by HPLC, and multiple PS modified pure diastereomers can be obtained by synthesis methods (Stec and Wilk, 1994; Hara et al., 2020). In the following, several examples could be presented to describe the PS modification for enhancing the affinity between aptamers and targets.

Liu et al. previously screened an adipocyte-specific aptamer named Adipo8 (Liu et al., 2012). However, two shortages including short half-life and low affinity hampered the therapeutic application of aptamers (Wang et al., 2011). To solve these mentioned above issues, Chen et al. used chemical modification approaches to modify aptamer Adipo8 by incorporating phosphorothioate linkages and conjugating polyethylene glycol (PEG) (Chen et al., 2015). Compared with unmodified Adipo8, they found that PS modified aptamer showed higher binding affinity to the target and performed high specificity *in vitro*. Furthermore, PS modified aptamer Adipo8 inhibited effectively adipogenic differentiation of adipose tissue, which showed potential in treating obesity.

Glypican-3 is the cellular membrane proteoglycan and is regarded as a tumor biomarker for diagnosing hepatocellular carcinoma. Recently, Dong et al. used capillary electrophoresis (CE)-SELEX technology to successfully screen an aptamer named AP613-1 with a 59.85 nM K_D_ value that can specifically bind to glypican-3 (Dong et al., 2018). When a phosphorothioate linkage was incorporated into APS613-1, the binding affinity of APS613-1 was improved to 15.48 nM (Dong et al., 2018). In addition, APS613-1 with PS modification was conjugated with Alexa Fluor 750. This conjugate could use for subcutaneous hepatocellular carcinoma imaging *in vivo*, indicating that it might be a potential agent in glypican-3 positive tumor imaging for diagnosing hepatocellular carcinoma.

Aptamer with PS modification also was used in detecting heavy metal cadmium ions, and it is exposure resulted in serious health risks. Recently, Yu screened an aptamer (CBA15) to develop a fluorescence anisotropy (FA) sensor for detecting cadmium ions. However, the CBA15 demonstrated unmet binding affinity to the target, which showed low sensitivity. To acquire better sensing performance, they used PS modification approach to modify aptamer’s phosphate backbone to improve the binding affinity of an aptamer to target by adding extra interaction between sulfur and cadmium ions. Compared with unmodified CBA15, PS modified CBA15 with a 47 nM K_D_ value represents a more than four-fold enhancement (Yu and Zhao, 2022).

#### 2.3.2 Phosphorodithioate (PS2)

The incorporation of phosphorodithioate (PS2) (Figure 6B) in the aptamers could also increase its binding affinity to the target proteins. Oligonucleotide (ODN) dithiophosphates are chemically modified ODN in which two sulfurs replaced non-bridging oxygen atoms at phosphate moiety (Volk and Lokesh, 2017). This modification strategy was used for improving the binding affinity of nucleic acid drugs and resist nuclease degradation. For example, it is usually used in aptamer modification for aptamer-based diagnostics and therapeutic applications. Abeydeera et al. showed that one dithiophosphate incorporation at a specific position could enhance binding affinity about ∼1000-fold for an RNA aptamer (Abeydeera et al., 2016). The synthesis of nucleoside dithiophosphates can be performed *via* several approaches. The most commonly used approach is the phosphoramidite approach (Beaucage, 1993). The one-pot reaction with H_2_S and tetrazole was prepared to construct phosphordithioate. The solid-phase synthesis of aptamers with PS2 modification is similar to traditional methods for synthesizing ODN. For the incorporation of phosphorodithioate, the conventional order of oxidation after capping was replaced by sulfurization before capping. This procedure could lead to fewer by-products.

The PS2 modified aptamers can resist the degradation of nucleases. This property had been evaluated by several labs (Sakatsume et al., 1992; Cummins et al., 1996). These findings pointed out that PS2 linkages in oligonucleotides were not hydrolyzed by nucleases (Murata et al., 1990). However, it had been reported that PS2-aptamers were as susceptible to DNase I as the natural aptamers (Ghosh et al., 1993).

The improved binding affinity of the PS2-ODNs to the target proteins has drawn some attention. Researchers have evaluated the interactions of the PS2 modified ODNs with proteins. Cheng et al*.* evaluated the interactions between *E. coli* single-stranded DNA binding protein (SSB) and ODNs with chemical modifications including PS2 modification (Cheng et al., 1997). The results demonstrated that the binding affinity of PS2-ODN to SSB was significantly higher than unmodified ODN. Additionally, the length of PS2-ODN could also influence the binding to SSB. Another team compared the cellular pharmacology of phosphorothioate and PS2-ODNs in HL60 cells (Tonkinson et al., 1994). They also demonstrated that PS2-ODNs could bind rsCD4 and bFGF to inhibit the activity of protein kinase C (PKC). In 2002, a team reported that the affinity of duplex aptamers targeting NF-κB was dramatically increased when the backbone was substituted by dithioate (Volk et al., 2002).

Because of the enhanced affinity and higher stability of the PS2 backbone-modifications, the thioaptamers have a promising future. A novel bead-based thioaptamer selection protocol was reported, which could be used to generate potential thioaptamers targeting specific proteins (Yang et al., 2003; Yang and Gorenstein, 2004). Hayes et al. demonstrated XBY-S2 thioaptamer targeting AP-1 with resisting nuclease degradation (Hayes and Salvato, 2012). Its antiviral ability against the West Nile virus has been tested. Further, the activity of XBY-S2 has been proven effective in animal models.

### 2.4 Extended alphabet (artificial nucleotides)

In the nature SELEX library, the types of nucleotides are limited (only dA, dT, dC, dG for DNA, or A, G, C, U for RNA), and these nucleotides have similar chemical structures, resulting in low chemical diversity. In contrast, there are 20 natural amino acids, which are made up of target proteins for aptamer. Therefore, increasing the chemical diversity of SELEX is regarded as a promising method to acquire high affinity aptamer (Hamashima et al., 2018).

**FIGURE 7:**
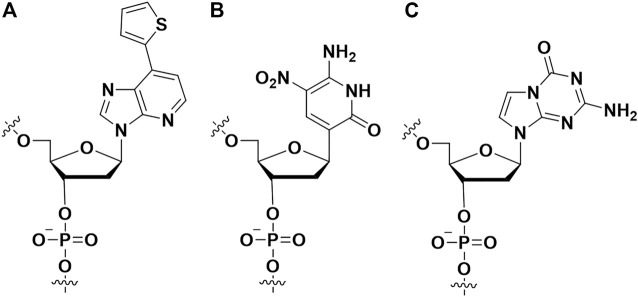
The artificial nucleotides incorporating into aptamer to improve binding affinity. **(A)** The (7- (2-thienyl) imidazo [4, 5-b] pyridine) (Ds) artificial nucleotide; **(B)** the 6-amino-5-nitro-3- (1′-β-D-2′-deoxyribofuranosyl)-2 (1H)-pyridone (Z) artificial nucleotide; **(C)** the 2-amino-8- (1′-β-D-2′-deoxy-ribofuranosyl)-imidazo-[1,2-a]-1,3,5-triazin-4 (8H)-one) (P) artificial nucleotide.

Hirao et al. first added artificial nucleotides into the SELEX library for high affinity aptamer selection, and they called it ExSELEX (genetic alphabet expansion for systematic evolution of ligands by exponential enrichment (Kimoto et al., 2013; Kimoto et al., 2023). All of the oligonucleotides in the SELEX library included predetermined 1-3 highly hydrophobic unnatural bases Ds (7- (2-thienyl) imidazo [4, 5-b] pyridine) (Figure 7A), which could enhance the hydrophobic interactions between the aptamer and target proteins. Then they identified two DNA aptamers bind respectively vascular endothelial cell growth factor-165 (VEGF-165) (K_D_ = 0.65 p.m.) and interferon-γ (IFN-γ) (K_D_ = 0.038 nM). The affinities of the aforementioned two aptamers are >100-fold enhanced over the aptamers which only contained natural nucleotides. Hirao’s group also improved the ExSELEX in a subsequent study, in which they constructed a Ds-randomized library improving the complexity of the initial version library (Matsunaga et al., 2017). Using the enhanced version ExSELEX, they identified the aptamer targeting von Willebrand factor A1-domain (vWF) (K_D_ = 75 p.m.). In addition, Hirao’s group also developed the mini-hairpin DNA to modify aptamer for improving the stability of unnatural-nucleotides aptamers without declining their affinity (Matsunaga et al., 2015; Kimoto et al., 2016). Benner’s group constructed a laboratory *in vitro* evolution system including A, G, C, T, Z (Figure 7B) and P (Figure 7C) (Biondi et al., 2016). They generated an aptamer binding protective antigen (PA) PA63 with a dissociation constant of ∼35 nM. Furthermore, by combining cell engineering technology and a laboratory *in vitro* evolution system, Benner and his colleagues found a series of aptamers including unnatural nucleotides targeting glypican 3 (GPC3) which was expressed on the surface of liver cells (Zhang et al., 2016). Artificial nucleoside incorporation could increase the complexity of the SELEX library, and it made aptamers more like proteins, thereby allowing aptamers to bind target proteins with high affinity. Recently, the Hachimoji eight-letter DNA/RNA was reported, which might promote significantly the aptamer field in the future (Hoshika et al., 2019).

## 3 Precision strike, new driving force for aptamer affinity improvement by chemical modification

The current researches put forward higher requirements for aptamers - precision strike, in which the target of the aptamer is not no longer the whole molecule but a part of it. Therefore, high affinity and specificity is required. For this purpose, chemical modification must be the most powerful sword. Recent works from Ichiro Hirao’s group succeeded to identify four dengue non-structural protein 1 (DEN-NS1) serotypes from clinical samples using expended alphabet Ds included aptamers (K_D_: 27–182 p.m.). The specificity of each aptamer is remarkably high, and the aptamers can recognize the subtle variants of DENNS1 with at least 96.9% amino acid sequence identity, beyond the capability of serotype identification (69–80% sequence identities (Matsunaga et al., 2021a; Matsunaga et al., 2021b). The presence of several Ds bases in these aptamers significantly increased the affinities and specificities to each target. Not alone, Ge Zhang’s group identified an aptamer, named aptscl56, which specifically targets the structural domain loop3 of sclerostin to promote bone formation without cardiovascular risks. Because the loop2 of plays a protective role in cardiovascular system, once the aptamer binds to it, it will lead to cardiovascular risk just like the previously reported monoclonal antibody. In their aptamer, four methoxy modifications at each 5′and 3′terminals not only provided nuclease-resistant ability, but also enhanced affinity (Yu et al., 2022). Following these concepts, it will be a trend to explore subdomain binding aptamers, in which chemical modification will definitely play his role well.

## 4 Discussion and future perspectives

The affinity of aptamers to the target protein is a key factor for their pharmacodynamics. Generally, the higher binding affinity was linked to better pharmacodynamics. The reduced dosage could contribute to less toxicity. The naturally occurring aptamer are composed of four kinds of nucleosides (A, G, C, T or U). Typically, naturally occurring aptamers show a decreased binding affinity than cognate antibodies. To enhance the affinity of aptamers, chemical modifications have great potentials to optimize the interactions between the aptamer and its ligand protein. Until now, many high affinity aptamers were generated by using chemical modifications either in pre-SELEX or post-SELEX procedures (Table 1). In the pre-SELEX procedure, the chemically modified nucleotide analogs were involved early in the construction of the SELEX library through phosphoramidite chemistry. Importantly, it has reached a conclusion that the involved nucleotide analogs must be compatible with DNA/RNA polymerase during PCR procedure. The next challenge is to understand which nucleotide analogs need what kind of DNA/RNA polymerase to maximize the PCR yield for improving the success rate of aptamer screening. Just like SOMAmers, the hydrophobicity of amino acid like side chains were demonstrated to have great potential in increasing hydrophobic interactions between the aptamers and target proteins (Vaught et al., 2010; Rohloff et al., 2014). Although chemical modifications benefit aptamers with abundant diversity and preferred nuclease resistance, there is rare evidence on which types of chemical modifications have the most impact. Accordingly, most chemical modifications hindered the integrity of SELEX libraries during their construction and the enrichment of high-affinity sequences, as well as the final sequencing procedure. In the post-SELEX procedure, the choice of chemical modifications is usually unrestricted, which significantly widens the diversity of modified aptamers. Additionally, chemical modifications in post-SELEX are site-specific. It indicates that diverse modifications can be simultaneously incorporated into aptamers to meet the criteria for optimizing properties. On contrary to pre-SELEX modification, pre-SELEX modification becomes complex and costly in the screening procedure. Not only did many sites were needed to be considered in the initial preparation of the modified aptamers, but also the screening method was limited. For example, depending on the researchers’ will if we want to manipulate 2 types of modification groups on a 40-nt long DNA aptamer, a total of 3^40^ possibilities needed to be considered, which cannot be completed by manpower. One of the shortcuts is to consider only one modification at one site of a known aptamer. Just as phosphorodithioate modification in an RNA aptamer, Xianbin Yang’s group tested every phosphorodithioate linkage at a single site and finally obtained ∼1000-fold of affinity enhancement in a case (Abeydeera et al., 2016). Additionally, structure-guided post-SELEX optimization is strongly recommended after a deep understanding of the interactions between the aptamer and its target (Xu et al., 2019). Last but not the least, high-throughput screening methods, such as DNA microchips, artificial intelligence, and combinational chemistry, could be involved to increase the capacity of post-SELEX modification. The possibilities of the modification types and modification sites in aptamer are both huge. It is impossible to characterize all the interactions of the modified aptamers to the target manually. Thus, it is necessary to seek an efficient virtual prediction strategy to optimize the modification types and modification sites of the aptamers for high binding affinity. Artificial intelligence (AI) has witnessed successes in predicting the interactions between targets and ligands in drug discovery as previously reported. Using high-throughput DNA chip technology, the binding ability data of aptamers with some modifications at some sites could be obtained. Based on the above data, the researchers could further build and train AI models to predict the enormous binding affinity of aptamers with different modifications at different sites.

**TABLE 1:** Chemically modified aptamers for improving binding affinity.

Target	Aptamer (length)	Modification strategy	K_D_	Ref
platelet-derived growth factor B	SL1 (29)	SOMAmer	0.02 nM	Davies et al. (2012)
Interleukin-8	8A-35 (35)	2′-fluoro-pyrimidine	1.72 pM	Sung et al. (2014)
HIV-1 reverse transcriptase	FA1 (77)	2′-deoxy-2′-fluoroarabinonucleotide	4 pM	Alves Ferreira-Bravo et al. (2015)
Salivary α-amylase	AMYm1 (75)	base-appended base	<1 nM	Minagawa et al. (2017)
vascular endothelial cell growth factor-165	AF83-7 (24)	PS2 modification	1 pM	Abeydeera et al. (2016)
Interferon-γ	IFd1-3Ds-49 (49)	Artificial Nucleotide Ds	0.038 nM	Kimoto et al. (2013)
vascular endothelial cell growth factor-165	VGd1-2Ds-47 (47)	Artificial Nucleotide Ds	0.65 pM	Kimoto et al. (2013)
